# MRI Monitoring of Cerebral Blood Flow after the Delivery of Nanocombretastatin across the Blood Brain Tumor Barrier

**DOI:** 10.4172/2157-7439.1000516

**Published:** 2018-10-25

**Authors:** Sunalee Gonawala, Madhava Aryal, James R. Ewing, Ana C. deCarvalho, Steven Kalkanis, Meser M. Ali

**Affiliations:** 1 Department of Neurosurgery, Cellular and Molecular Imaging Laboratory, Henry Ford Hospital, USA; 2 Department of Neurology, Henry Ford Hospital, Detroit, MI 48202, USA

**Keywords:** MRI, Dendrimer, Nanocombretastatin, Cerebral blood flow, Brain tumor

## Abstract

Introduction of polymeric nanoparticles in cancer therapeutics is widely investigated since nanomedicine often enables the intratumoral delivery of drugs with increased efficacy with minimal side effects. In this study MRI monitoring was employed to study the therapeutic effect of nanocombretastatin (G3-CA4) in an orthotopic glioma model. Water insoluble combretastatin (CA4) was conjugated to a small-sized water soluble G3-succinamic acid PAMAM dendrimer. Nanoconstruct sizes were determined by TEM to be 3 to 5 nm. Intravenous (i.v.) delivery of G3-CA4 in an orthotopic glioma model produced a long-lived ischemia accompanied by necrosis at the core of the tumor but leaving a rim of viable tissue. In contrast, delivery of CA4 alone has no therapeutic effect in an experimental rat model of glioma.

## Introduction

Glioma is a malignant central nervous system neoplasm that is highly vascular and resistant to all current therapies. High-grade gliomas, while relatively rare [1,2] are almost invariably fatal; even given best therapy, the median survival is about 15 months [3]. These tumors have proven very difficult to treat. Cancer treatment usually begins with surgery followed by radiation or chemotherapy [4].

Increased vascularization and angiogenesis are fundamental to glioma cell survival and proliferation [5]. Malignant gliomas are typically characterized by a discharge of vascular endothelial growth factor (VEGF), which is a crucial regulator and promoter of angiogenesis [6]. As a result, anti-angiogenic remedies targeting VEGF or VEGF receptors (VEGFRs) were proposed as effective tools for regulating the growth of malignant gliomas. However, early clinical trials using humanized monoclonal antibodies against small-molecule tyrosine kinase inhibitors targeting different VEGFRs and VEGF (Bevacizumb) alone or together with other therapeutic agents showed mixed results [6–8]; most reports indicate the gliomas developed resistance to anti-angiogenic medications [7–10].

Alternative therapies known as Tumor-Vascular Disrupting Agents (T-VDAs) diverge from anti-angiogenic strategies and directly target established tumor vasculature. T-VDAs selectively disrupt the immature and rapidly proliferating endothelial cells [11,12] of established tumor vasculature by direct apoptosis or by effects associated to the endothelial cell’s reliance on a tubulin cytoskeleton to preserve cell shape. These agents block the blood flow in tumors, resulting in an ischemia leading to a cascade of secondary tumor cell death in the tumor core [13,14]. T-VDAs can have drastic effects on the three dimensional shape of freshly formed endothelial cells, with slight or no consequence on quiescent endothelial cells [14,15] because newly formed endothelial cells are more sensitive to treatment than mature cells, since the former lack a fully developed actin cytoskeleton that maintains cell shape despite depolymerization of the tubulin cytoskeleton [16,17]. The most promising T-VDA is combretastatin (CA4), [18–20] though water-solubility poses a major challenge. Combretastatin phosphate (CA4-P) which is a water-soluble form of CA4 has been synthesized to improve bioavailability. CA4-P also has the ability to disrupt cell-cell contacts between endothelial cells mediated by the vascular endothelial (VE)-cadherin/β-catenin complex [21]. However, the presence of smooth muscle cells, a characteristic feature of normal tissue vasculature, inhibits this disruption [22]. Targeting newly formed endothelial cells in immature or abnormal vessels that lack a full complement of smooth muscle or pericyte support is believed to contribute to drug specificity [14,17]. Although CA4 can cause necrosis in the tumor core of various types of solid tumors [17,23], in gliomas CA4 alone does not effectively induce a central necrosis.

Tumor vasculature disruption approaches are suitable for nanoparticle-based therapeutics because they do not require the drug to be delivered to every cell in the tumor. We hypothesize that nanoformulation of CA4 may represent an improved approach to target tumor vasculature of glioma. The typically high levels of angiogenic activity in brain tumors results in the formation of abnormally dilated vessels with fenestration and leaky inter-endothelial gaps [24–26]. In this hyper-permeable vasculature nanoparticles can extravasate and be preferential retained in the tumor interstitium following systemic administration, a phenomenon known as the Enhenced Permeability and Retention (EPR) effect [27] albeit the effective transvascular delivery of nanoparticles through the blood-brain tumor barrier (BBTB) of malignant gliomas remains a challenge. A recent study to evaluate the effect of nanoparticle size on BBTB permeability demonstrated that gadolinium chelated dendrimer nanoparticles with core sizes less than 12 nm penetrated the BBTB, whereas larger nanoparticles were restricted [27–30]. Spherical nanoparticles with core sizes between 4 to 10 nm that are smaller than the upper limit of pore size in the BBTB retained peak blood concentrations for several hours [27,31,32]. Dendrimer-based paramagnetic nanoparticles that can preferentially accumulate in an orthotopic preclinical glioma model were recently developed [26,33].

While there are nanoformulations of CA4 developed for the distribution of the active form of CA4 to tumors [34], their sizes have been significantly larger than 12 nm, the upper limit of pore size in the BBTB [27,31]. With this in mind, we proposed to use CA4 conjugated to our recently developed dendrimer-based nanoparticle (G3-CA4) with nanoscale diameters (3 to 8 nm) via a hydrolytically labile ester linkage for the development of an effective brain drug delivery system, and to follow the delivery and action of this nanoparticle with noninvasive MR imaging. We hypothesized that the EPR effect of these dendrimer-based G3-CA4 would lead to an accumulation in brain tumors and induce necrosis at the primary GBM core.

## Materials and Methods

All reagents used were purchased from Sigma Aldrich (St. Louis, MO) unless otherwise stated. G3-PAMAM succinamic acid (10 wt. %) in water was purchased from Sigma Aldrich and dried in a freeze dryer prior to use. 4-Dimethylaminopyridine (99%), Dimethylsulfoxide anhydrous (≥ 99.9%), N,N’-dicyclohexylcarbodiimide (99%) and Combretastatin-A4 (≥ 99) were used without further purifications. Dendrimeric combretastatin conjugates were purified by repeated ultrafiltration with deionized water using appropriate molecular weight cut-offs (Millipore’s Amicon Ultra centrifugal filters). ^1^H-NMR spectra were obtained using a VARIAN MR 400 NMR spectrophotometer. Deuterated DMSO (DMSO-d_6_) was used as NMR solvent. Chemical shifts (δ) are given in ppm with reference to the internal standard Tetramethylsilane (TMS, δ=0 ppm). The molecular weights of the conjugates were analyzed using ESI-Mass or MALDI-TOF spectrometry.

### Synthesis of G3-PAMAM-(Succinamic acid)-Combretastatin_26_ conjugate

To a solution of G3-PAMAM-(succinamic acid)_32_ (0.15 g, 0.015 mmol, 1 equiv.) dissolved in DMSO (15 ml), combretastatin-A4 (**CA4**) (0.15 g, 0.48 mmol, 32 equiv.) dissolved in DMSO (10 ml) was added. Then to the above reaction mixture, 4-dimethylaminopyridine (1.0 mg) and N, N’-Dicyclohexylcarbodiimide (0.099 g, 0.48 mmol, 32 equiv.) were added and the reaction was stirred for 24 h at room temperature. After 24 h, the reaction mixture was transferred to a 500 ml Erlenmeyer flask, diluted with distilled water (400 ml) and stirred at room temperature for 0.5 hour. After this, the reaction mixture was left in refrigerator overnight to allow the precipitate to settle. The resultant clear solution was filtered through 0.45 μm filter unit. Finally, the filtrate was diafiltered using Amicon^®^ Ultra centrifugal filter (3,000 MWCO, regenerated cellulose) and was washed with distilled water (3 times) under the same conditions. The sample was then pre-frozen in liquid nitrogen and freeze dried. Finally, 0.16 g of white product was obtained. MALDI-TOF (Figure S1) m/z: 18500 g.

### Animal model

Animal studies were performed adhering to approved procedures of the Institutional Animal Care and Use Committee of Henry Ford Hospital, Detroit, MI. U-251 human glioma cells were provided by Dr. Ana C. deCarvalho, Henry Ford Hospital. These cells were cultured in 75 cm^2^ tissue culture flasks using Dulbecco’s modified Eagle’s medium (DMEM) supplemented with 10% fetal bovine serum (FBS), penicillin (100 IU/mL), and streptomycin (100 μg/mL) until they were 80–90% confluent. The cells were collected by trypsinization, washed and centrifuged to make a cell suspension of 4 × 10^5^ cells/5 μL.

Six- to eight-week-old athymic nude rats weighing 150–170 g (Charles River Laboratory, Inc.) were anesthetized by intraperitoneal injection of a mixture of 100 mg/kg ketamine and 15 mg/kg xylazine. Each rat was placed on a stereotactic head holder (Kopf, Cayunga, CA), the surgical zone was shaved and swabbed with betadine solution, and the eyes were coated with Lacri-lube (Allergan Inc., Irvine CA). After draping, a 1-cm incision was made 2 mm to the right of the midline, 1 mm retro-orbitally and the skull was exposed with cotton-tip applicators. An HP-4 dental drill bit was used with a micromanipulator to drill a hole 3 mm to the right and 1 mm anterior to the bregma, with care not to penetrate the dura. A #2701 10 μL Hamilton syringe with a #4 point, 26 s gauge-needle containing 4 × 10^5^ U-251 tumor cells in 5 μL was lowered to the depth of 3.5 mm, then raised to the depth of 2.5 mm. The cells were injected stepwise at a rate of 0.5 μL/30 sec until the entire volume was injected. During and after the injection, careful note was made for any reflux from the injection site. Two to three minutes after completing the injection, the syringe was withdrawn in a stepwise manner. The surgical hole was sealed with bone wax. Finally, the skull was swabbed with betadine before suturing the skin over the injection site. The orthotopic tumor was allowed to grow for 4 to 5 weeks to a diameter of 3 to 5 mm.

### G3-PAMAM-(Succinamic acid)-Combretastatin_26_ pharmacokinetics in serum: LC-MS/MS method for determination of CA4 in serum

The concentrations of CA4 in serum were estimated by mass spectrometric detection (LC-MS/MS). To 100 μl of plasma, 200 μl methanol containing 2% formic acid was added, and the mixture was vortex-mixed for 1 minute, then centrifuged at 14000 RPM for 2 minutes. Zileuton was used as internal standard. The supernatant was collected, and a volume of 10 μl was injected into the HPLC. Chromatographic analysis was performed using a Waters Model 2695 separations system (Milford, MA, USA) as described in our previous report [35].

### *In vivo* MRI and measurement of Cerebral blood flow (CBF)

All MRI studies were performed using a 21 cm bore, 7 Tesla magnet and a Bruker spectrometer and console running Paravision 6.0.1. Gradient maximum strengths and rise times were 250 mT/m and 120 μs. All MRI image sets were acquired with a 32 × 32 mm^2^ FOV. The transmit coil was a RAPID (RAPID MR International, Columbus, OH) Quadrature Birdcage coil; the receive coil was RAPID two-channel phased-array surface coil for rat brain imaging.

About 22 days after tumor implantation each animal was anesthetized (0.8% to 1.0% halothane) and a 26 g dental catheter was inserted into a tail vein to allow the injection of contrast agent. The animal was then placed in the magnet bore. Core temperature was controlled at (37°C ± 1°C) using a warm-air supply. Arterial spin labeling (ASL), pre- and post-contrast T_1_-weighted, pre-contrast T_2_-weighted, and pre-contrast apparent diffusion coefficient (ADC) image sets were acquired. Prior to the second T_1_-weighted image, a bolus injection of the CA (Magnevist, Bayer Healthcare Pharmaceuticals, Wayne, New Jersey), 0.25 mmol/kg at undiluted concentration, no flush, was performed by hand push.

Tissue perfusion was imaged using arterial spin labeling MRI (ASLMRI) using a fast spin-echo acquisition [36]. Perfusion imaging time was ~13 min with the following parameters: TR =1500 ms, 16 Segments, 4 echoes per segment, echo spacing = 12.02 ms, NEX = 8, Matrix = 128 × 64, Spectral Width = 25 kHz, FOV = 32 mm, Slice = 1 mm. After a locater image, high-resolution T_2_, and a pre-treatment flow sequence was run, and then either CA4 (50 mg/kg) or G3-CA4 (50 mg/kg, equivalent to CA4 dose) was administered via an indwelling catheter.

Prior to drug administration, a pulsed gradient spin-echo DWI sequence was run in three directions (x, y, z) to generate a parametric map of the trace of ADC. DWI sequence parameters were as follows: matrix 128 × 64, 13 slices, 0.8 mm thickness, 0.2 mm gap, repetition time (TR) =1500 ms, echo time (TE) = 40 ms, number of echoes (NE) = 1, b-values = 0, 600, 1217 s/mm^2^, gradient amplitude=107 mT/m, gradient duration = 10 ms).

Two high-resolution T_1_-weighted spin-echo images were acquired pre- and post-CA, to locate the tumor and its size, with the following parameters: FA = 45°, 180°, matrix 256 × 192, 27 slices, 0.4 mm thickness, 0.1 mm gap, NE = 1, NA = 4, TE/TR = 16/800 ms. A high-resolution T_2_-weighted spin-echo image was acquired pre-contrast with the following parameters: FA = 90°, 180°, matrix 256 × 192, 27 slices, 0.4 mm thickness, 0.1 mm gap, NE = 4, NA = 2, TE/TR = (20, 40, 60, 80)/3000 ms).

### Histopathology

Tissues were stained for red with vWF-TRITC and green for FITC-tomato lectin delineating endothelial lining and blue for nuclear dapi using standard immune histochemical procedure as described in our previous report [36].

## Results and Discussion

### Synthesis

**Figure 1:**
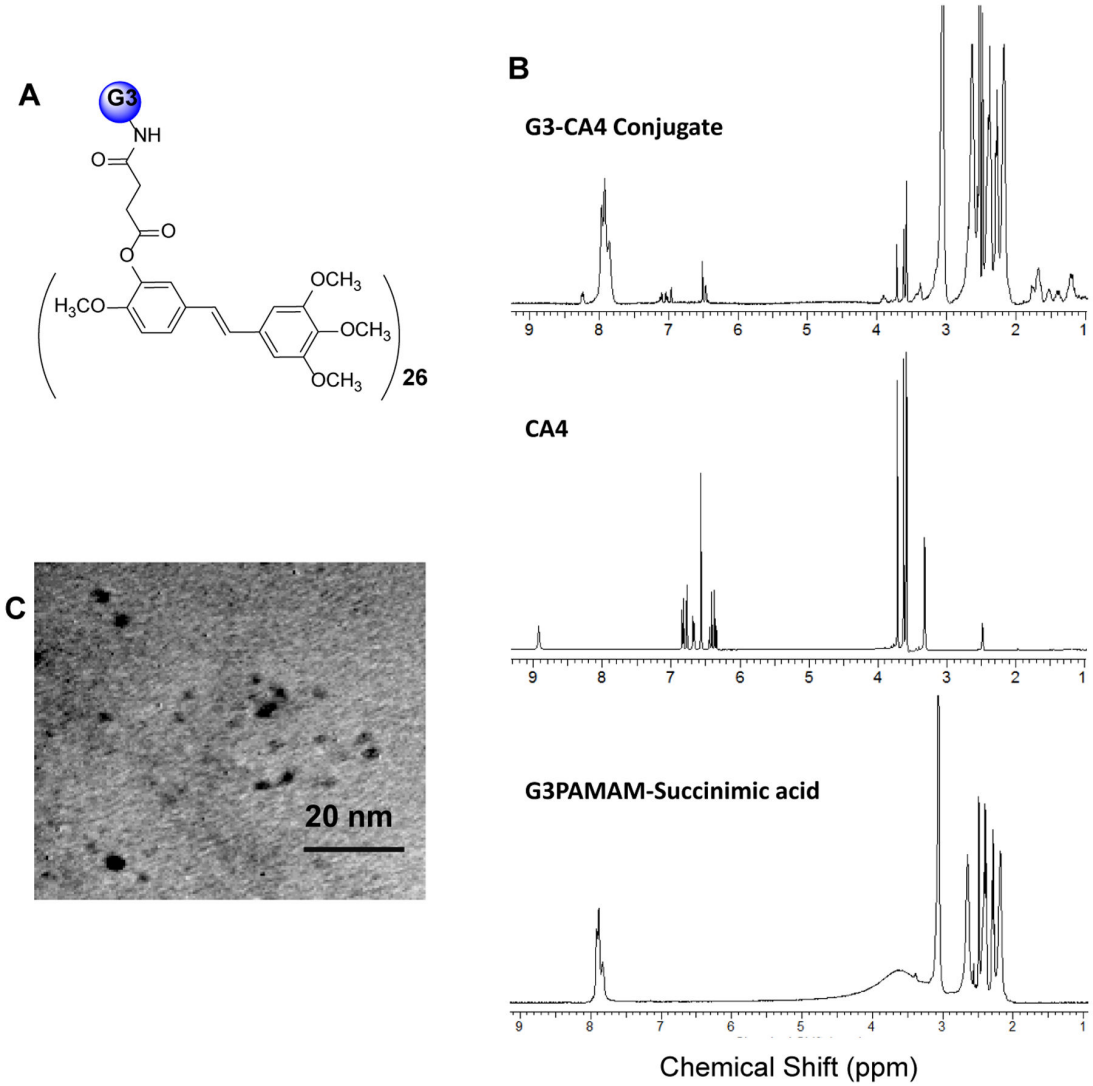
Schematic view of CA4 conjugated with a G3 PAMAM succinamic acid dendrimer (A). ^1^H NMR spectrum of G3-(CA4)_26_, CA4, and G3 PAMAM succinamic acid in DMSO (B), TEM image of G3-CA4 conjugate (C).

The DMAP (4-dimethylaminopyridine)/DCC (dicyclohexylcarbodiimide) coupling method was used to conjugate CA4 with G3-succinamic acid dendrimer (G3-COOH)_32_. The G3-CA4 conjugate (Figure 1A) was purified by diafiltration using a C-3 membrane filter (MWCO 3000) and characterized by ^1^H-NMR, UV-visible, and MALDI-TOF mass spectrometric methods. The ^1^H-NMR spectrum Figure 1B showed multiplets between δ 1.0 and 4.0 ppm corresponding to the presence of -CH and -CH_2_ protons from G3-PAMAM-succinamic acid and CA4. The peaks between δ 6.4 and 8.4 ppm correspond to the presence of hydrogen atoms on the phenyl rings in CA4 [37]. The peak at δ 8.9 ppm in CA4 that corresponds to -OH is absent in the resulted G3-CA4 conjugated product due to the conjugation with G3-succinamic acid dendrimer. Due to the overlapping of the multiple peaks in the NMR spectrum, a complete characterization of the proton spectra was not achieved.

The number of CA4 molecules per dendrimer was estimated using MALDI-TOF mass spectroscopy. G3-succinamic acid (G3-COOH)_32_ has a mass of 10,111 g/mole and G3-CA4 conjugate was shown to have a mass of 18,500 g/mole (Figure S1). Since CA4 has an approximate mass of 316.34 g, the conjugate was shown Figure 1A to contain 26 CA4 molecules per dendrimer corresponding to a conjugation yield of 81%. The size of G3-CA4 conjugate was estimated to be approximately 3 to 5 nm using transmission electron microscope (TEM) images as shown in Figure 1C. The G3-CA4 polymer conjugate (Figure 1A) was highly water soluble, while natural free CA4 was water insoluble [38]. Therefore, we succeeded in conjugating a water insoluble CA4 with highly soluble G3 polymers that bear an additional advantage as a prodrug with enhanced aqueous solubility which in turn increase the bioavailability of the drug that will result in enhanced cytotoxic activity.

### Absorption spectra

**Figure 2:**
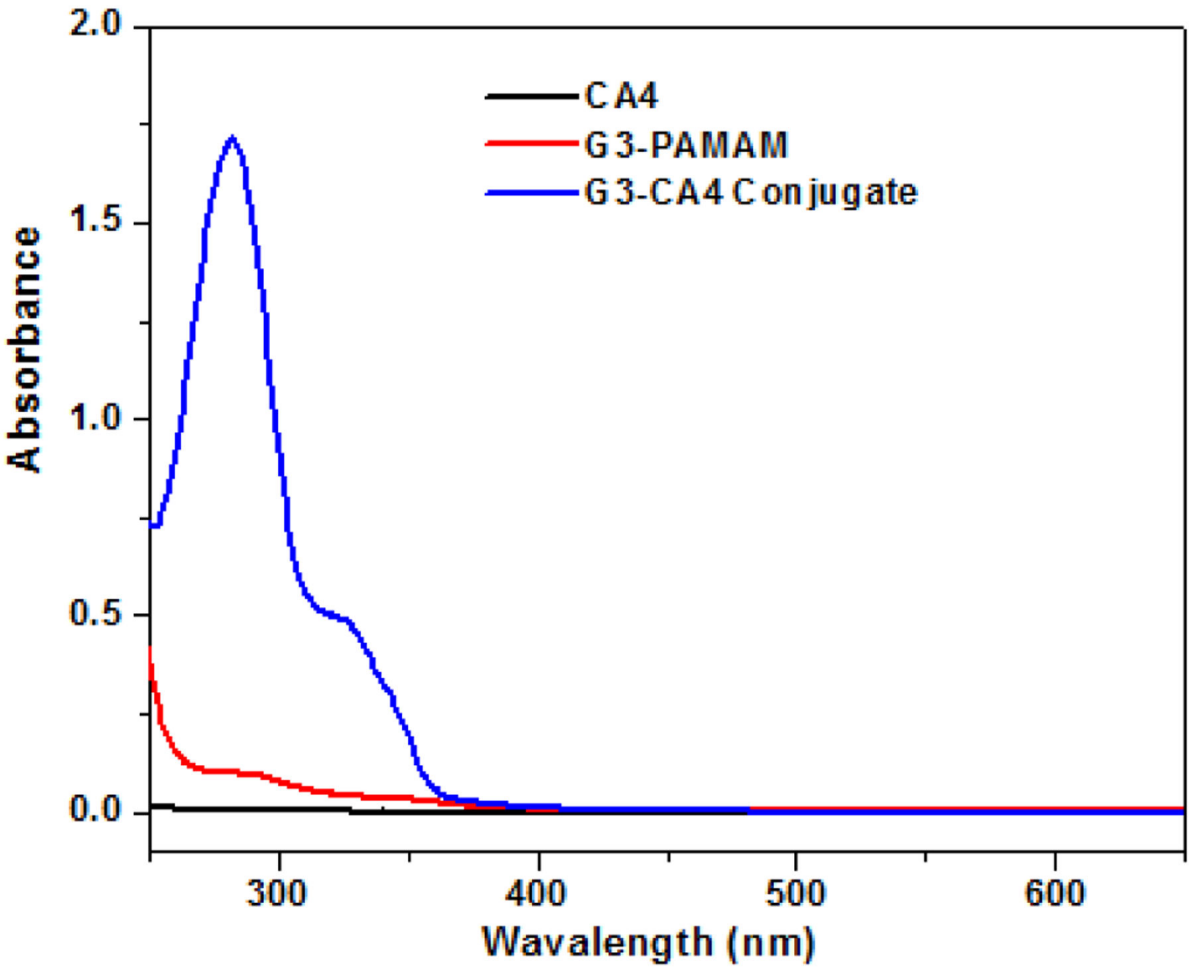
Absorption spectra of CA4 (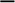) G3-PAMAM (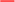) and G3-CA4 conjugate (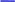) in water.

The absorbance spectra of CA4, G3-succinamic acid, and G3-CA4 conjugate (in H_2_O) is shown in Figure 2. Only G3-CA4 showed an absorption maxima (λ _max_) around 325 nm and 280 nm. Like G3-Cur, conjugation of CA4 to G3 dendrimer did not shift the absorption maxima of free CA4 [35]. Free CA4 in DMSO was used to generate a standard curve for the estimation of CA4 content in G3-CA4 conjugate as shown in Figure S2.

### Pharmacokinetics of the G3-CA4

All animal experiments were approved by the Institutional Animals Care and User Committee (IACUC). We determined serum pharmacokinetics of the G3-CA4. In order to perform bioavailability of G3-CA4, the rats were randomly assigned to either G3-CA4 or CA4 group. G3-CA4 (100 mg/kg equivalent to CA4 in water) was administered to rats intravenously. The rats were anesthetized at 0, 0.25, 0.5, 1, 2, 3 and 4 hours later, blood was collected by femoral catheterization, with the serum separated by centrifugation at 14000 rpm for 2 min. HPLC profiles of serum are depicted in Figure S3. 3 rats were used for each time point. A maximum serum concentration of 31 μM of G3-CA4 per mL was detected at 0.25 h after intravenous administration of G3-CA4. LC/MS/MS method for the detection and quantification of free CA4 in rat serum was also performed, and the amount of free CA4 in the serum was not detectable. Therefore, conjugation of CA4 to a G3 dendrimer resulted an improved bioavailability of CA4. However, CA4 levels in the serum reported was as CA4 released from G3-CA4 conjugate per milliliter of serum.

### In vivo MRI monitoring Cerebral Blood Flow in U251 cerebral tumors after treatment with CA4 and G3-CA4

Tumor blood flow differs from that of normal tissue [14], particularly in its lack of regulation. This leads to a vulnerability that can be exploited by agents like CA4 which weaken tubulin in the microvessels of the tumor, leading to the collapse of the microvessels and failure of perfusion in the tumor core.

**Figure 3:**
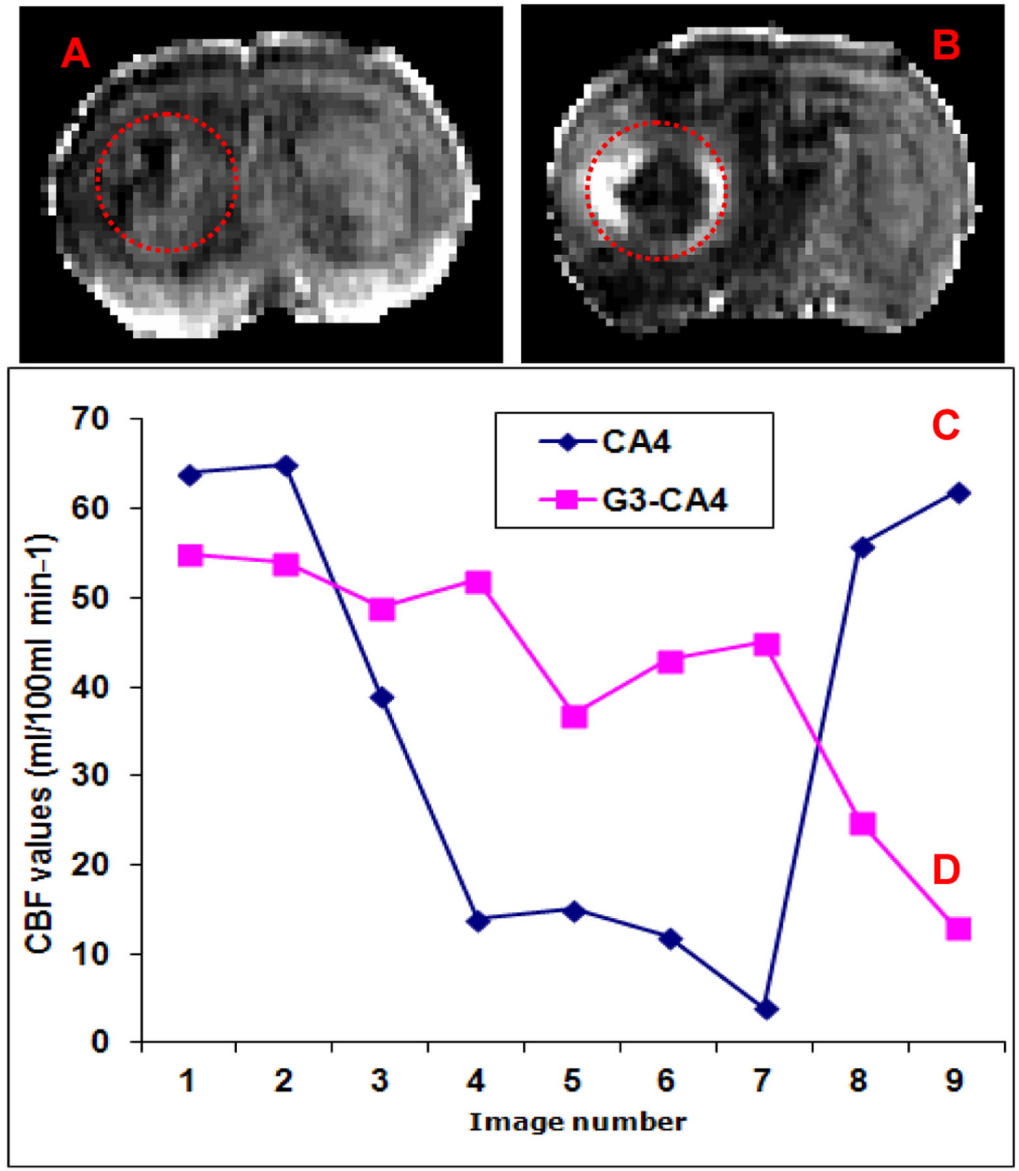
Representative CBF maps of CA4 (**A**) and G3-CA4 (**B**) treated rat brains bearing tumors after 24 hours post treatment. Blood flow vs time post-therapy in two nude rats with implanted U251 tumors. The first two points in each curve are flow pre-treatment. The last two points were taken ~ 24 h post-treatment. The five points following the first points were taken at ~20 min intervals. Blue curve (**C**) is flow post-CA4. The rapid decrease in flow, followed by a recovery at the 24 h point is evident. Pink curve (**D**) is flow post-G3-CA4 treatment. The long-term effect of the latter treatment is evident in the depressed flow at the 24 h points.

**Figure 4:**
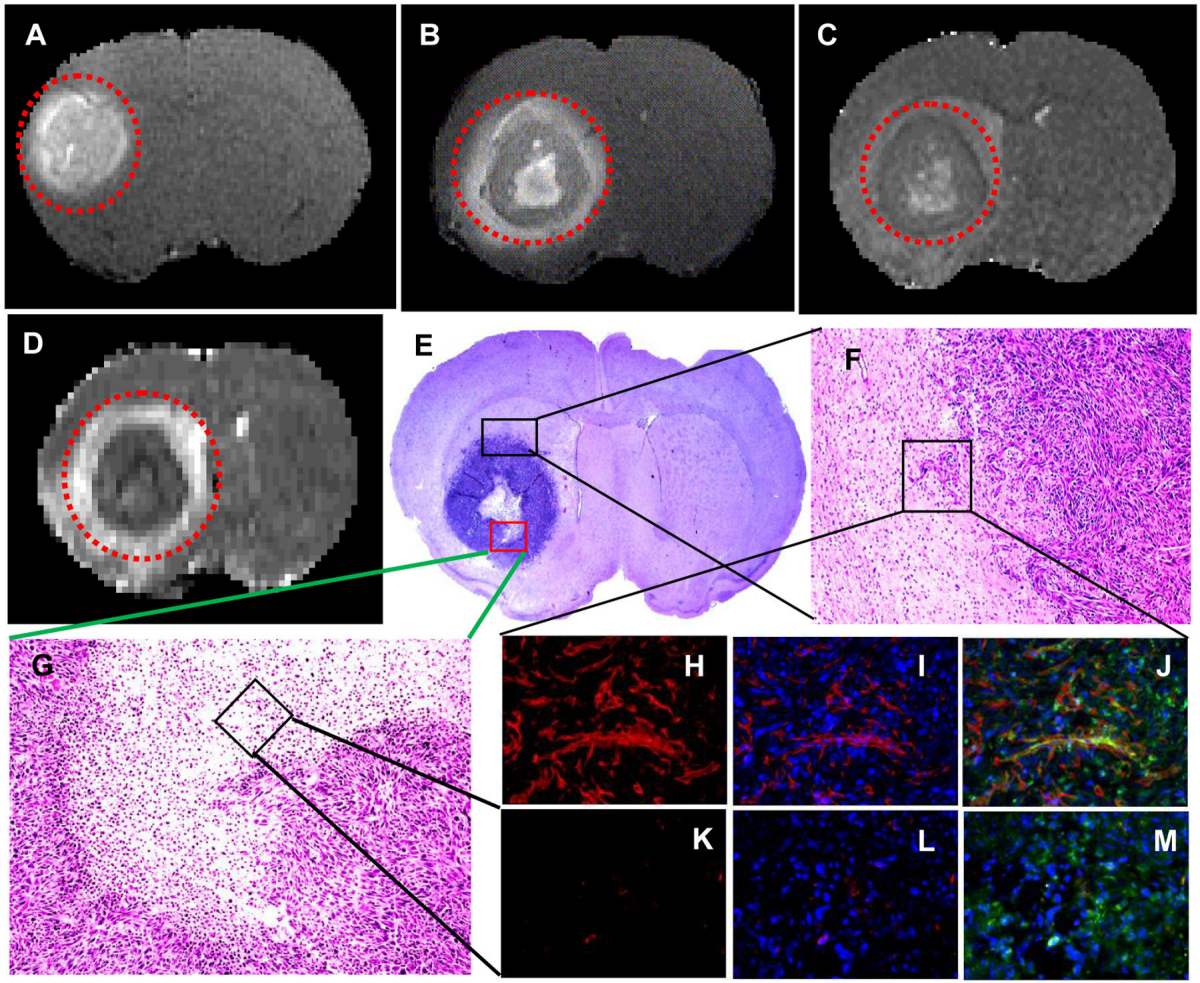
Post-contrast T_1_-weighted MR images 24 h after administration of G3-succinamic acid polymer as vehicle (A) and G3-CA4 (B). T_2_ (C) and ADC maps (D) after 24 h. Tumors are indicated as dotted red circles. Hematoxylin-eosin stain proves massive intratumoral necrosis (E & G) leaving viable tumor cells at the rim (F). Representative images of tumor blood vessels (red visualized with vWF-TRITC staining) at panels H, I & J at the rim. In contrast, the core of the tumor at panels K, L & M did not show any active blood vessel. Red for vWF-TRITC and green for FITC-tomato lectin delineating endothelial lining and blue for nuclear dapi.

Nude rats (n = 2) with intracerebral U251 tumors were studied 20 days after implantation. After a locator image, high-resolution T_2_, ADC, and two pre-treatment flow studies were acquired. Following this, either CA4 (50 mg/kg) or G3-CA4 (50 mg/kg, equivalent to CA4 dose) was administered via an indwelling catheter. Five more flow studies were conducted at ~20 min intervals followed by contrast agent injection and a post-contrast T_1_-weighted study. The animal was then removed from the magnet. Twenty-four hours later, the T_1_, T_2_, ADC, and two further flow studies were conducted. Please, see Figures 3C and 3D, where a clear difference in the recovery of flow can be observed in the two treatments; while flow after CA4 treatment exhibited a characteristic rebound, flow after G3-CA4 treatment remained depressed, indicating the continued effectiveness of the G3-CA4 treatment. The T_2_ (Figure 4C) and ADC (Figure 4D) maps show a ring of edema around a core of frank necrosis, signaled by a ring of increased T_2_ and ADC [39]. Neither the edema (data not shown), nor the necrosis, were observable in the pre-treatment MRI. Similarly G3-succinamic acid vehicle treated MRI also did not show any sign of edema or necrosis (Figure 4A, a post-contrast T_1_-weighted image). The animals were sacrificed and perfused [40] and tumors were excised and further processed & assessed using standard histopathology techniques. We investigated (by H&E staining of tumor sections) the effect of G3-CA4 upon the morphology of the tumors (Figure 4E). A Large necrotic area is found together with destroyed blood vessels at the core of the tumor leaving viable tissue at the rim of the tumor (Figure 4F and 4G). Endothelial lining in the tumor is clearly delineated by FITC (green) labeled tomato lectin (Figure 4J) and the tumor peripheral active blood vessels (Figure 4H) are stained by TRITC (red) labeled *Von Willebrand factor* (vWF). The nucleus of the cells is delineated by DAPI staining. Figure 4 (panels H to M) demonstrates the fluorescent microscopic images of tissue sections from tumor rim and core followed by G3-CA4 treatment. Use of vWF staining at the rim (Figure 4H) and the core (Figure 4K) of the tumor has allowed us to assess changes in vessel numbers in G3-CA4 treated tumors. The vascular shutdown effect and subsequent intratumoral necrosis caused by i.v. delivery of G3-CA4 is clearly observed in U251 tumor. This demonstrates that a vascular bed responsive to G3-CA4 can be identified by a blood flow measurement.

The ineffectiveness of small molecule chemotherapy drugs in treating GBM has been attributed to the BBB being a significant impediment to the transvascular extravasation of drug fraction across the barrier into the extravascular compartment of tumor tissue. The overall ineffectiveness of these approaches can be attributed to the fact that there is only a transient elevation in drug concentrations within the extravascular extracellular compartment of tumor tissue due to the short blood half-life of small molecule chemotherapy [27]. We have previously developed highly water soluble dendrimer-based paramagnetic nanoparticles [26,41–43] and demonstrated the relative pharmacokinetics of generation G2 and G5 dendrimer-based paramagnetic nanoparticles within xenografts models by noninvasive MRI [26,42]. G2-based particle showed deeper tumor penetration while G5-based particle accumulated at the rim of the tumor first and then moved to the other parts of the tumor [41]. Like G3-CA4, we have also conjugated dietary supplement, curcumin (Cur), a yellow pigment in the spice turmeric (Curcuma longa) with a G3 succinamic acid dendrimer in our previous report [35]. Cur exhibits green fluorescence and has structural similarity with CA4. Both Cur and CA4 are natural product hydrophobic compounds. In addition, the G3-Cur conjugate has same size and surface charge as G3-CA4. While systemic delivery of Cur leads to non-specific distribution throughout the body [44], G3-Cur conjugate preferentially accumulated in an orthotopic preclinical glioma model, minimizing systemic toxic effect [35]. An examination of tissue distribution in the same rat model showed that fluorescent labeled generation 3 (G3-Cur) was not present in major organs: heart, liver, spleen, lung and kidney [35]. We observed true success of delivering a significant amount of G3-Cur into brain tumor but not to the ipsi-lateral or contra-lateral normal brain regions. Thus, a G3-succinatecurcumin nanoparticle showed more accumulation in tumor and/or less renal uptake, possibly as a result of small particle size, neutral surface charge and hydrophilicity in blood. We have conjugated 24 curcumin molecules with G3-succinate as fluorescent dye. Similarly, we have also conjugated 26 molecules of CA4 with G3-succinate polymer. Since, G3-Cur_24_ [35] and G3-(CA4)_26_ (Figure 1) are closely related in size, charge and structure, we expect that they also display very similar *in vivo* pharmacokinetics and biodistributions; we expect that G3-CA4 selectively accumulates at the U251 tumor site, avoiding off-target distribution. We have demonstrated that systemic delivery of G3-CA4 induced a permanent blood flow shut down, resulting in cellular death at the core. In contrast, CA4 depressed blood flow transiently with flow returning 24 h post treatment. Intravenous (i.v.) delivery of G3-CA4 in an orthotopic human glioma model caused necrosis at the core of the tumor, as validated by histo-pathlological studies (Figure 4). We have shown in our previous studies [40] that the U-251 glioma tumor exhibits relatively more expression of hypoxia inducible factor-1α (HIF-1α) at the core of the tumor than that of tumor periphery. It has been long known that the hypoxic microenvironment markedly affects the response of tumors to various treatments such as chemotherapy, radiotherapy and hyperthermia. Therefore, G3-(CA4)_26_ causes the destruction of large areas of the interior of brain tumors that are typically resistant to conventional chemo- and radio-therapies. By applying the designed nanoprodrug strategy and tumor-specific prodrug activation mechanism, we observed the *first* true success of inducing necrosis at the core of U-251 glioma tumor. We have demonstrated that a tumor vasculature disrupting approach is particularly suited for nanoparticle based therapeutics with a compromised blood brain barrier. PAMAM dendrimers of G7 and lower are generally used in human and animal systems as multifunctional carriers because they closely mimic the size of biomolecules [45]. However, PAMAM-based dendrimers are not considered to be biodegradable polymers; in particular, the G3-CA4 conjugate is not a biodegradable construct. However, once its potential is demonstrated, a CA4 conjugate with a biodegradable G3 polymer can be constructed for clinical translation.

## Conclusion

Vascular disrupting agent, CA4 was conjugated with a G3-succinamic acid polymer. Conjugation of CA4 with G3 polymer improved high water solubility as well as bioavailability. TEM images showed that the sizes of G3-(CA4)_26_ nanoparticles were in the range of 3 to 5 nm, suggesting that the system could benefit from the EPR effect. The construct without CA4 conjugation had no therapeutic effect on glioma whereas, CA4 conjugated nanocombretastatin showed significant blood flow decrease in an experimental rat model of glioma. CA4 alone showed transient blood flow reduction, but the flow recovered after 24 hours. G3-(CA4)_26_ induced intratumoral blood vessels collapse leading to necrosis at the core of the tumor while the blood vessels at the periphery were alive. The delivery vehicle introduced here can be also loaded with other antiangiogenic drugs that can destroy active peripheral blood vessels, thus offering a possibility of combination therapy.

## Supplementary Material

Supplemental
