# Supplementary material for: MRI Monitoring of Cerebral Blood Flow after the Delivery of Nanocombretastatin across the Blood Brain Tumor Barrier
Source: J Nanomed Nanotechnol. Author manuscript; Available in PMC 2019 Jan 15. (PMC6333422; doi:10.4172/2157-7439.1000516)
Supplement: Supplemental [file NIHMS998735-supplement-Supplemental.pdf]

MRI Monitoring of Cerebral Blood Flow after the Delivery of Nanocombretastatin across the Blood Brain -Tumor Barrier

Sunalee Gonawala[1], Madhava Aryal, James R. Ewing[2], Ana DeCarvaloh[1], Steven Kalkanis[1] and Meser M. Ali[1]*

Supplementary materials:

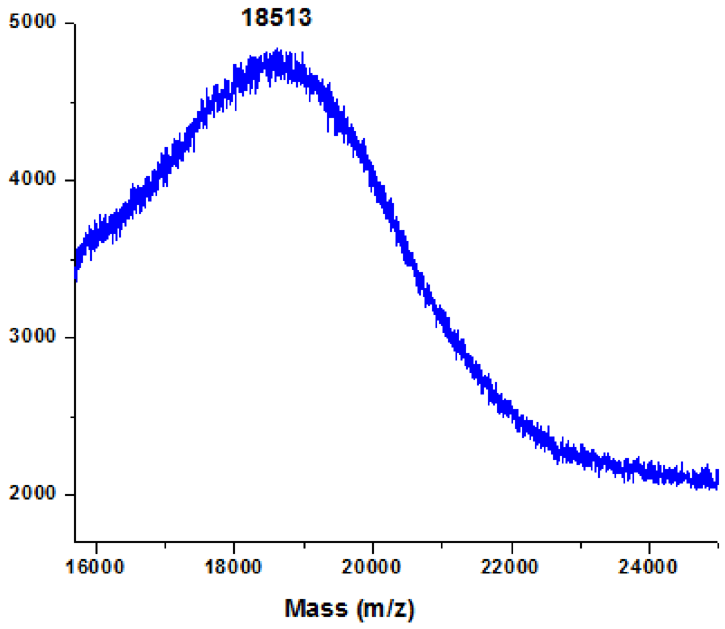

Figure S1: MALDI-TOF spectrum for molecular weight determination of G3-$(CA)_{26}$. The average number of CA4 conjugated with G3-succinamic acid dendrimer was estimated to be 26 per dendrimer.

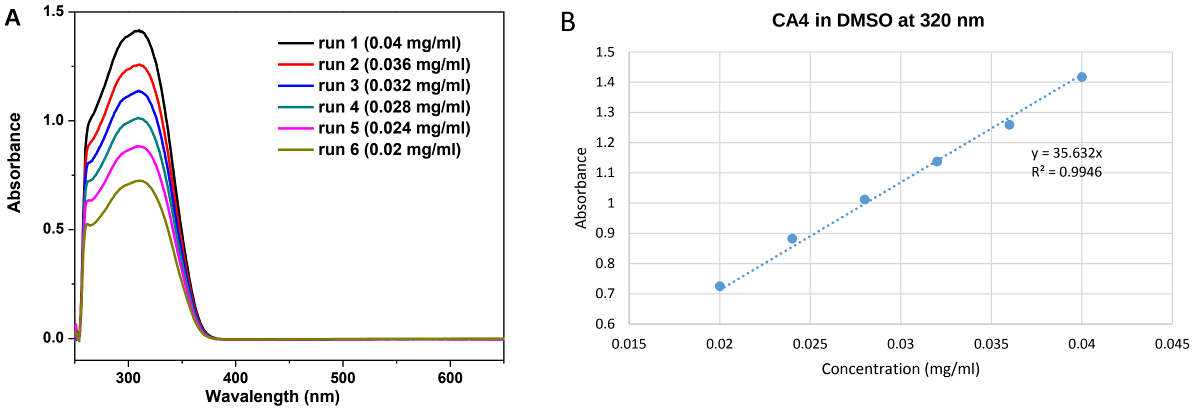

Figure S2: Absorption spectra of G3-CA4 in water (A), standard curve for CA4 in DMSO (B).

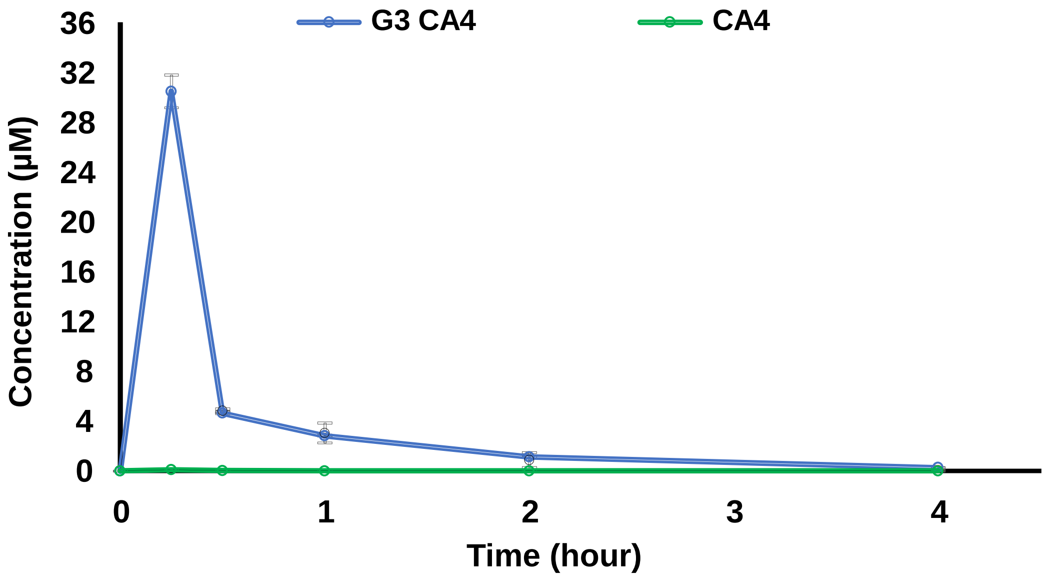

Figure S3: Bioavailability of G3-CA4 in rat. G3-CA4 (100 mg/kg) was administered to rat by intravenously. The rats were anesthetized at 0, 0.25, 0.5, 1, 2, 3 and 4 hours later, blood was collected by femoral catheterization, with the serum separated by centrifugation at 14000 rpm for 2 min. HPLC analysis of serum are depicted in the figure.
